# Response-Predictive Gene Expression Profiling of Glioma Progenitor Cells *In Vitro*

**DOI:** 10.1371/journal.pone.0108632

**Published:** 2014-09-30

**Authors:** Sylvia Moeckel, Katharina Meyer, Petra Leukel, Fabian Heudorfer, Corinna Seliger, Christina Stangl, Ulrich Bogdahn, Martin Proescholdt, Alexander Brawanski, Arabel Vollmann-Zwerenz, Markus J. Riemenschneider, Anja-Katrin Bosserhoff, Rainer Spang, Peter Hau

**Affiliations:** 1 Department of Neurology and Wilhelm Sander-NeuroOncology Unit, University Hospital Regensburg, Regensburg, Germany; 2 Institute for Functional Genomics, University of Regensburg, Regensburg, Germany; 3 Department of Neurosurgery, University Hospital Regensburg, Regensburg, Germany; 4 Department of Neuropathology, Regensburg University Hospital, Regensburg, Germany; 5 Institute of Pathology, University of Regensburg, Regensburg, Germany; Medical University Graz, Austria

## Abstract

**Background:**

High-grade gliomas are amongst the most deadly human tumors. Treatment results are disappointing. Still, in several trials around 20% of patients respond to therapy. To date, diagnostic strategies to identify patients that will profit from a specific therapy do not exist.

**Methods:**

In this study, we used serum-free short-term treated *in vitro* cell cultures to predict treatment response *in vitro*. This approach allowed us (a) to enrich specimens for brain tumor initiating cells and (b) to confront cells with a therapeutic agent before expression profiling.

**Results:**

As a proof of principle we analyzed gene expression in 18 short-term serum-free cultures of high-grade gliomas enhanced for brain tumor initiating cells (BTIC) before and after *in vitro* treatment with the tyrosine kinase inhibitor Sunitinib. Profiles from treated progenitor cells allowed to predict therapy-induced impairment of proliferation *in vitro*.

**Conclusion:**

For the tyrosine kinase inhibitor Sunitinib used in this dataset, the approach revealed additional predictive information in comparison to the evaluation of classical signaling analysis.

## Introduction

High-grade gliomas include the most frequent type of primary central nervous system (CNS) tumors, glioblastoma (GBM) [1]. GBM show diffuse infiltration of the surrounding brain, making a curative surgical resection impossible. Moreover, GBM are molecularly heterogeneous. Consequently, the clinical management of GBM is challenging and outcomes are poor with a median survival time of only 14.6 months [2], and only rare long-term survivors [3].

Sunitinib is a small-molecule inhibitor (SMI) with antiangiogenic and antitumor activity mediated through inhibition of multiple receptor tyrosine kinases (RTKs). Sunitinib is FDA-approved for the first-line treatment of advanced renal cell carcinomas and progressive gastrointestinal stromal tumors (GIST) resistant to Imantinib [4], [5]. Interestingly, Sunitinib shrinks renal cell carcinoma CNS metastasis in the brain [6], suggesting that it crosses the blood-brain-barrier (BBB). The prevalent VEGF receptors 1–3 and platelet derived growth factor receptor (PDGFR) α/β are targets of Sunitinib [7]. Their role in GBM growth and neovascularisation has been widely studied [8], [9]. In preclinical studies using *in vitro* models of primary CNS tumors Sunitinib inhibited proliferation and migration [10]–[14]. Particularly in GBM models, Sunitinib alone [15] or in combination with radiation therapy [16] showed potent antiangiogenic and antiinvasive effects. Clinical trials of Sunitinib and other small-molecule inhibitors targeting RTKs have however shown a clinical response only in subgroups [1], [17], [18] of GBM patients. Disappointingly, the amplification and mutation status of the targeted receptors alone are not predictive for response [1], [18].

The cancer stem cell hypothesis postulates that clones of pluripotent cells exerting stem cell like properties [19], [20] induce tumors and maintain the tumor bulk. This would make them the primary targets of treatment [21]. Brain tumor initiating cells (BTICs) are propagated under serum-free conditions, undergo sustained self-renewal and retain tumorigenic potential forming tumors that recapitulate the phenotypes of parental tumors [22]–[25]. Existing literature suggests that this subpopulation of tumor cells holding stem-cell like features contribute to chemotherapy resistance [26]. Regarding response to radiation conflicting results have been reported [27], [28]. Consequently, these cells might hold relevant information for predicting therapy response.

Today, treatment decisions for GBM patients are based on age, performance status [29], and increasingly on molecular markers like *MGMT* promoter methylation. Recent genomic studies established sub-classifications of GBMs based on gene expression profiling [30], [31] or integrated genetic and epigenetic profiling [32]. These GBM subtypes were associated with distinct prognosis and benefit from classical chemo-radiotherapy. No specific treatment selection including novel targeted agents can be derived from these classifications.

Here, we suggest to use expression profiles of *in vitro* treated tumor cell cultures to predict treatment response. As a first development step towards this approach, we treated 18 short-term cultures of high-grade gliomas with Sunitinib. To sharpen predictive expression patterns we enriched specimens for brain tumor initiating cells (BTIC). From these specimens we generated expression profiles before and 6 hours after treatment, and signatures for treatment response were constructed to predict *in vitro* proliferation and migration after treatment.

## Materials and Methods

### Tumor samples and patient characteristics

Native glioma tissue samples were obtained from patients undergoing surgical resection at the local Department of Neurosurgery with a diagnosis of high-grade glioma WHO grade III or IV. All tumors were histologically classified according to the 2007 WHO classification of tumors of the central nervous system by the local neuropathologist (MJR). Specimens were cultured according to current criteria for the culture of brain tumor initiating cells (BTIC) [22]. In addition to conventional histology, GFAP and IDH1 (R132H) immunoreactivity as well as *MGMT* promoter methylation (by methylation specific PCR) were assessed in the primary operation material, and the same parameters plus Nestin (by Western blot) were repeated in the short-term BTIC cultures. Clinical data of all patients were followed until disease progression, and overall survival was evaluated using the RANO criteria [33]. All patients gave written informed consent, and this study and further use of the samples were specifically approved by the ethics committee of the University of Regensburg, Regensburg, Germany (No° 11-103-0182).

### Primary cell culture of brain tumor initiating cells (BTICs)

Tissue samples were kept in PBS at 4°C and processed within 24 hours after surgery. Samples were mechanically dissociated using a scalpel followed by aspiration through a Pasteur pipette. If cells did not dissociate spontaneously, enzymatic dissociation with 1% Trypsin/EDTA at 37°C for 5 minutes maximum was performed. After washing with PBS, cells were passed through a cell strainer with 30 µm pore size to obtain a single cell suspension (Merck Millipore, Darmstadt, Germany). Remaining tumor cells were cultured in stem-cell permissive RHB-A media (Stem Cell, Cambridge, UK) supplemented with 20 ng/ml of each human recombinant epidermal growth factor (EGF; R&D Systems, Minneapolis, USA) and human recombinant basic fibroblast growth factor (FGF; Peprotech, Hamburg, Germany). Culture media were replaced by fresh media with the indicated supplements twice a week. Under these *in vitro* conditions BTIC specimen grew either as spheres or exhibited adherent growth spontaneously (Table S1). To verify tumor-initiating capacities of our BTIC primary samples, some cultures were transplanted orthotopically in immunocompromised mice (data not shown). In addition, stem cell marker expression was documented by immunohistochemical staining for Nestin and Sox2 and flow cytometry analysis of CD133 expression (partly presented in Table S1). Differentiation capacity was confirmed by immunohistochemical staining for differentiation markers of specific neural lineages (GFAP, GalC, βIII-Tubulin) after cultivation in 10% FCS for 14 days (not shown). Clonogenicity was tested in a 96-well single cell dilution assay (not shown). The lowest available passage of all BTIC primary cultures (usually below passage 8) was used for all assays.

### Treatment of BTIC cultures with Sunitinib

Sunitinib was purchased from Sigma Aldrich (St. Louis, Missouri, USA) and prepared as a 25 mmol/l stock solution in aliquots of 0.5 ml in DMSO for *in vitro* studies. BTICs were grown in cell culture dishes (TPP, Trasadingen, Switzerland) until they formed a subconfluent monolayer (density of 80%). Laminin coated dishes were used for cells that grew non-adherent under stem cells conditions (Table S1). Before treatment, cells were cultured in growth factor free medium for 16 hours to simulate *in vivo* conditions. After starvation, cells were treated with 1 µM Sunitinib in the treatment groups or 0.00025% DMSO in the control groups either with or without supplementation of recombinant growth factors PDGF-A/B and VEGFA (25 ng/ml) for 6 hours before harvest. Each treatment combination was set up twice. Cells were either harvested in RLT-lysis buffer (provided in the RNeasy Kit, Qiagen, Hilden, Germany) for subsequent RNA-extraction or in lysis buffer (25 mM Tris, 150 mM NaCl, 5 mM EDTA, 10% Glycerol, 1% Triton X100, 10 mM Na-Pyrophosphat, 1 mM Na-Orthovanadate, 10 mM Glycerol phosphate) for whole cell protein extraction.

### Microarray analysis

Hybridization to arrays was performed in the local Competence Center for Fluorescent Bioanalytics. Quality of RNA was confirmed by HPLC and RNA was further processed by reverse transcription. cDNA was converted to Biotin-labeled cRNA which was the hybridized to Affymetrix hugene.1.1.st GeneChips (Affymetrix, Santa Clara, California, USA). Data are deposited at the gene expression omnibus (GEO) functional genomics data repository under accession number GSE51305.

### RT- and Quantitative PCR

First-strand specific cDNAs were generated by using the reverse transcription kit (Promega, Madison, USA). Quantification of mRNA expression was performed by real-time PCR (Mx3000P Quantitative PCR [qPCR] System, Stratagene, Germany) using the Brilliant III Ultra-Fast SYBR Green QPCR Ultra fast Master mix (Agilent Technologies, Santa Clara, California, USA). A standard curve with serially diluted cDNA was prepared for the target gene and reference gene (β-Actin). Primer sequences are listed in the supplementary section (Table S2).

### Western blot analysis

For Western blot analysis, 15 to 20 µg of total cell lysates were diluted in 15 µl of Laemmli buffer, separated on a 10% SDS-PAGE gel and transferred to nitrocellulose membranes by semi-dry blotting. The membranes were blocked with 5% milk powder in 0.02% Tween TBS (TBST) for 1 hour. Based on previous results on key molecules regulated by Sunitinib (Pfizer, investigators brochure, March 2008), membranes were incubated with antibodies against Akt, phospho-Akt (Ser473), STAT3, Phospho-STAT3 (Tyr705) (all from Cell Signaling, Danvers, USA), ERK1/2, phospho-ERK1 (T202/Y204)/ERK2 (T185/Y187) (all from R&D Systems, Minneapolis, USA), and β-Actin (Sigma Aldrich, Missouri, USA) or GAPDH (Santa Cruz Biotechnology, Heidelberg, Germany) overnight at 4°C. Bound antibodies were visualized with a horseradish peroxidase–linked antibody against mouse or antibody against rabbit immunoglobulin G (R&D Systems, Minneapolise, USA) followed by enhanced chemiluminescence reaction (Roche Applied Science, Basel, Switzerland). Western blots were repeated three times.

### Proliferation assay

BTICs were grown in a 96-multiwell plate and treated with 1 µM Sunitinib in the treatment group or 0.00025% DMSO in the control group, diluted in stem cell media supplemented with PDGF-A/B and VEGFA (each 25 ng/ml) for indicated times. Non-adherent cells were seeded on Laminin-coated wells. XTT Reagent was added to the media 4 hours prior to the photometric measurement, and cellular viability was assessed by the Cell Proliferation Kit II (XTT) from Roche Applied Science (Roche, Basel, Germany) according to the manufactures protocol. Photometric evaluation was performed with the Varioscan ELISA reader (Thermo Scientific, Massachusetts, USA). For every individual BTIC line the XTT assay was repeated at least three times. Proliferation rates were calculated for each treatment, and values were prepared for correlation analysis to microarray and other data performed by bioinformatics as described below.

### Migration assay

The formation of round-shaped aggregates of BTIC cells *in vitro* typically occurs in a non-adherent microenvironment. Therefore, so called multicellular tumor spheroids were generated by plating 100 µl of a single cell suspension (3–5×10^4^/ml) on an agar-coated well. Mature spheroids with a mean area of 0.45 mm^2^ were explanted to round-bottom 96-well plates containing the corresponding treatment (1 µM Sunitinib in the treatment group or 0.00025% DMSO in the control group) diluted in stem cell media supplemented with 25 ng/ml of the recombinant growth factors VEGFA and PDGF-A/B. Transferred spheroids were monitored using a light microscope. Cells were allowed to migrate from the spheroid surface for 16 hours. Spheroid areas were measured using the ImageJ Software (NIH, Bethesda, USA), and spreading of surfaces was evaluated in 8 spheroids of each treatment group. Median surface areas were calculated for each treatment, and absolute levels of expansion were recorded. For further correlation analysis spheroid areas were corrected for size differences at time point zero.

### Computational Analysis and Statistics

Computational analysis was performed using R and Bioconductor (http://www.bioconductor.org). Expression values were corrected and normalized using RMA [34]. To account for the data structure we compensated for between patient variability in a pre-processing step using the bioconductor package ComBat [35] by modeling every patient as a separate “batch” of four samples (before/after treatment; with/without growth factor). Differentially expressed genes were identified using the package Limma [36]. Limma is based on linear models. We included growth factor treatment of cell cultures into the models to adjust for its confounding effect on gene expression. False discovery rates (FDR) of gene lists were calculated according to Benjamini Hochberg [37]. Signatures for quantitative prediction of treatment response were learned using ‘Least Angle Regression’ [38] as implemented in the bioconductor package LARS. Note that LARS analysis incorporates the selection of signature genes. Prediction performance was validated in leave one out cross validation. Selection of signature genes was done separately for all left out samples.

If not specified otherwise statistical analysis of *in vitro* data was performed using the student’s t-test. A p-value less than 0,05 was considered to be statistically significant (*, p<0,05; **p<0,01, ***p<0,001).

## Results

### Characterization of patient material

Eighteen native glioma tissue samples were obtained from patients undergoing surgical resection at the local Department of Neurosurgery. Tumors were neuropathologically classified as GBM in 16 cases, gliosarcoma in 1 case and anaplastic astrocytoma in another case (Table S1). *MGMT* promoter methylation varied from 0 to 100%, and 2 cases were mutated at the R132H locus. MGMT promotor methylation was followed in the short-term BTIC cultures (Table S1).

### Sunitinib induced modulation of signaling pathways can be detected for therapeutic dosages of Sunitinib and can be boosted by supplementary growth factors

**Figure 1:**
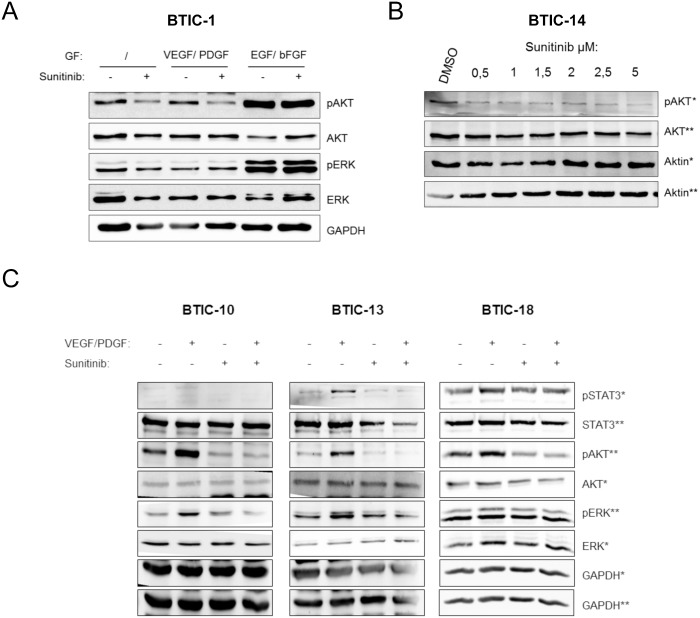
Phosphorylation pattern of signaling molecules downstream of Sunitinib target receptor tyrosine kinases. Western Blot analysis was performed with 18 BTIC lines of which 3 representatives are shown. (A) To evaluate distinct phosphorylation patterns under treatment, BTIC-1 was treated with 1 µM Sunitinib or 0.00025% DMSO for 6 hours with growth factor supplementation (25 ng/ml) as outlined. (B) To evaluate a dose curve for Sunitinib, BTIC-14 cells were incubated with different Sunitinib-concentrations or 0.001% DMSO in media supplemented with 25 ng/ml of each VEGFA and PDGF-AB. (C) After definition of growth factor supplementation and Sunitinib dose, Western Blot analysis for changes in phosphorylation after treatment with Sunitinib was performed with 18 BTIC lines of which 3 representatives are shown. Cells were treated with 1 µM Sunitinib or 0.00025% DMSO with (+) or without (–) growth factors (GF) PDGF-AB and VEGFA (25 ng/ml) for 6 h after incubation in growth factor free medium for 16 h. The asterisks (*) indicates the corresponding loading control. GAPDH was used as protein loading control.

Brain tumor initiating cells from high-grade glioma resections were starved in serum- and growth factor-free medium prior to treatment. After treatment with various concentrations of Sunitinib we assessed the activity of potential downstream signaling cascades by Western blot with or without growth factor supplementation (Fig. 1A). Growth factor supplementation was used to examine the inhibition of pre-stimulated pathways. Growth factor free conditions were chosen to investigate the blockage of autocrine and paracrine pathway activation. VEGF/PDGF, but not EGF/bFGF activated signaling was considerably abolished by Sunitinib. Sufficiently strong responses were observed when cell cultures were activated with 25 ng/ml PDGF-A/B and VEGFA and cells remained without further exogenous stimuli during Sunitinib treatment.

Next, the Sunitinib dosage was calibrated using a concentration row (Fig. 1B). A concentration of 0.5 µM of Sunitinib was sufficient to abolish PDGF/VEGF-stimulated activation of AKT while still lying in the range of concentration measured in tumor tissue of patients treated with Sunitinib [39], [40].

### Heterogeneous treatment response is mirrored in Sunitinib induced modulation of signaling pathways

All 18 BTIC lines were treated with 1 µM of Sunitinib or 0.00025% DMSO as control. Next, phosphorylation of downstream signaling molecules of Sunitinib target-RTKs pSTAT3, pAKT and pERK1/2 were assessed by Western blots (Fig. 1C and Table 1). Signal intensities in reference to the related unstimulated samples were evaluated by 3 independent investigators for each BTIC line yielding semi-quantitative consensus strengths of response. The response to treatment *in vitro* was again heterogeneous. Phosphorylation status of STAT3 and AKT were lower in Sunitinib treated cells than in controls in 64% and 70% of evaluated signals without prior VEGF/PDGF-stimulated activation. The strength of inhibition varied considerably. Inhibition of ERK-phosphorylation was observed in only 35% of cases although and was less pronounced than STAT3 and AKT inhibition (Fig. 1C and Table 1). Further, the induction potential on the STAT3, AKT and ERK pathways by VEGF/PDGF differed considerably.

**Table 1:** Phosphorylation pattern of signaling molecules after treatment.

Protein		BTIC																	
		1	2	3	4	5	6	7	8	9	10	11	12	13	14	15	16	17	18
+ GF	pSTAT3	**+**	**+**	**++**	**+**	**0**	**0**	**++**	**0**	**++**	**0**	**0**	**+**	**+**	**+**	**nS**	**0**	**++**	**+**
	pAKT	**+**	**0**	**++**	**+**	**0**	**+**	**+**	**0**	**+**	**++**	**+**	**+**	**++**	**+**	**++**	**++**	**++**	**++**
	pERK1/2	**+**	**0**	**0**	**0**	**0**	**+**	**0**	**+**	**+**	**+**	**0**	**0**	**+**	**0**	**++**	**0**	**0**	**+**
**+ Sunitinib**	pSTAT3	**– –**	**– –**	**– –**	**– –**	**u.e.**	**–**	**–**	**–**	**0**	**n.S.**	**–**	**–**	**0**	**0**	**n.S.**	**u.e.**	**0**	**0**
	pAKT	**0**	**–**	**–**	**–**	**–**	**0**	**–**	**–**	**0**	**–**	**–**	**0**	**–**	**–**	**–**	**u.e.**	**0**	**–**
	pERK1/2	**0**	**– –**	**0**	**0**	**0**	**–**	**–**	**0**	**0**	**0**	**–**	**–**	**0**	**0**	**–**	**u.e.**	**0**	**0**
SunitinIb + GF	pSTAT3	**– –**	**– –**	**– –**	**– –**	**u.e.**	**– –**	**– –**	**–**	**– –**	**n.S.**	**–**	**0**	**– –**	**–**	**n.S.**	**u.e.**	**– –**	**0**
	pAKT	**– –**	**–**	**– –**	**–**	**– –**	**–**	**– –**	**–**	**–**	**– –**	**–**	**–**	**– –**	**– –**	**– –**	**0**	**– –**	**–**
	pERK1/2	**–**	**– –**	**0**	**–**	**0**	**–**	**–**	**–**	**–**	**– –**	**–**	**+**	**–**	**0**	**– –**	**+**	**0**	**0**

To evaluate changes in the phosphorylation of Sunitinib targets, cell lines were treated with growth factors (GF; 25 ng/ml of hPDGF-AB and hVEGF-A), 1 µM Sunitinib, or a combination of Sunitinib plus growth factor in 18 BTIC lines of high-grade gliomas. Changes in the phosphorylation intensity of pSTAT3, pAKT and pERK1/2 compared to the untreated control sample were graded as follows by three independent investigators: ++ strong increase, + moderate increase, 0 no change, – moderate decrease, – – strong decrease. Some samples were unevaluable (u.e.) or showed no signal (n.S.).

In summary, we observed heterogeneous responses to Sunitinib treatment on the levels of pathway activation, signal propagation, and target protein expression. This result underscores the need to identify responders prior to treatment.

### Sunitinib inhibits proliferation in a subset of cases

**Figure 2:**
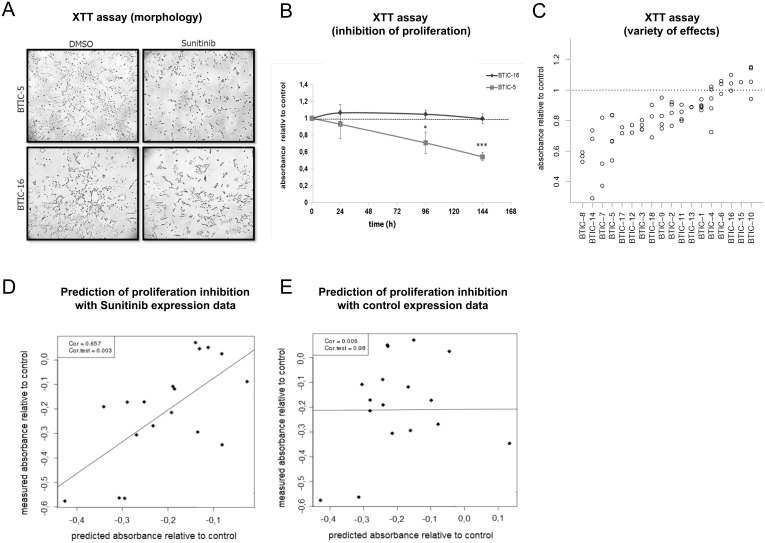
Cellular growth and proliferation under Sunitinib treatment. BTICs were incubated with 1 µM Sunitinib or 0.00025% DMSO (control), and the XTT proliferation assay was performed after 96 h. Each individual assay was performed with five replicates per treatment group. The assay was repeated at least three times for each cell line. (A) Growth pattern in a responding (BTIC-5) and a non-responding (BTIC-16) BTIC line. Representative pictures are shown for two differently responding BTIC lines. (B) The mean absorbance of Sunitinib treated cells relative to control cells obtained in an individual assay was assessed after 24 h, 96 h and 144 hours incubation period and is plotted in a dot blot graph (y-axis) against incubation time (x-axis). (C) The relative difference of the mean proliferation relative to control is blotted in a dot blot graph (y-axis) against the corresponding BTIC line (x-axis). Each data point indicates the result of an individual experiment. The assay shows the variety of effects in the investigated lines. (D) Prediction of proliferation based on gene expression 6 h after treatment in vivo. The x-axis shows cross validated predictions of proliferation response after 96 hours based on gene expression levels monitored 6 hours after treatment, while the y-axis shows the actual proliferation measurements after 96 hours. The correlation between predicted and measured proliferation is significant (p<0.01, chi-square test). (E) Failed prediction of proliferation using expression values from untreated samples. There is no significant correlation between predictions and measurements (p = 0.98).

We assessed the proliferation of all 18 BTIC lines at several time points between 24 and 144 hours after Sunitinib treatment by XTT assays and compared them to control samples observed over the same time period. Again, different responses were observed (Fig. 2A, B).

For 60% of the BTIC, treatment for 24 hours had no detectable effect (data not shown). However, after 96 hours, the number of viable cells was clearly reduced in responding BTIC lines (Fig. 2B, p = 0.005). This trend was even more pronounced at later time points.

Following this preliminary analysis, a 96 hour XTT assay was repeated at least three times for each BTIC line (Fig. 2C). We observed inhibition of proliferation of up to 56% in 3 BTIC lines, whereas 4 lines exhibited no detectable response at all. BrdU assays were performed for 2 of the BTICs to control for XTT assay validity yielding similar proliferation inhibition rates as the XXT assays (Fig. S1). Further we proved that PDGF/VEGF preserves constant proliferation capacity of BTICs for the analyzed time span (Fig. S2, p = 0,1). Again the observed heterogeneity underscores the need to identify responders prior to treatment.

### Sunitinib inhibits cellular motility in a subset of cases

**Figure 3:**
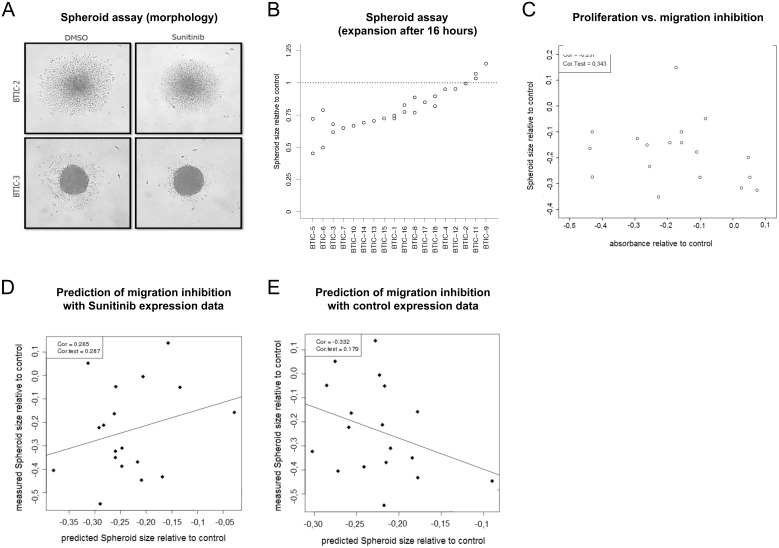
Migration under Sunitinib examined by multicellular spheroid expansion assay. Spheroids were incubated with 1 µM Sunitinib or 0.00025% DMSO (control) in a round-bottomed 96 well plate. Radial expansion of BTICs from the spheroid was recorded after 16 hours incubation. (A) Representative pictures of two differently responding BTIC lines (BTIC-2 and BTIC-3) show different patterns of migration. (B) The relative difference of the mean covered area relative to control is blotted in a dot blot graph (y-axis) against the corresponding BTIC line (x-axis). (C) The mean spheroid size relative to control (y-axis) is blotted against the corresponding relative proliferation inhibition (x-axis) for each of the 18 BTIC lines. (D) Failed prediction of migration on gene expression 6 h after treatment in vivo. The x-axis shows cross validated predictions of migration, while the y-axis shows the actual migration measurements. There is no significant correlation between predictions and measurements (p = 0.287). (E) Failed prediction of migration using expression values from untreated samples. There is no significant correlation between predictions and measurements (p = 0.179).

A tumor spheroid-based migration assay that simulates tumor cell dissemination from a solid microtumor [41], [42] was used to investigate tumor cell motility after treatment with 1 uM Sunitinib or control. In this assay, tumor cells propagate from the spheroid over the dish surface, and migration rates in terms of an area covered by radial spreading were measured using area calculation by ImageJ 16 hours after treatment. A pair of exemplary BTICs in which migration was differentially inhibited is shown in Fig. 3A. The relative difference of spreading areas under treatment with Sunitinib in comparison to control was calculated for each individual BTIC line (Fig. 3B). In line with all previous observations responses varied substantially across BTIC lines. To validate these data, we performed wound migration assays in BTIC cultures. All cultures evaluated with both methods showed comparable migration inhibition rates (Fig. S3). Interestingly, inhibition of proliferation and inhibition of migration did not correlate (Fig. 3C, p = 0.343), suggesting that Sunitinib targets these phenotypes via distinct signaling pathways. Once more, the observed heterogeneity underscores the need to identify responders prior to treatment.

### Heterogeneity of signaling pathway activation results into genome wide expression profiles

Transcriptome wide expression profiles of BTIC enriched cell cultures were generated before and 6 hours after *in vitro* treatment with Sunitinib using Affymetrix hugene.1.1.st GeneChips. RNA samples from Sunitinib treated BTICs were collected concurrently with protein samples. All 18 BTIC lines were treated with 1 µM Sunitinib or 0.00025% DMSO with and without the combination of VEGF and PDGF-AB for 6 hours after overnight starvation in serum- and growth factor-free medium.

**Figure 4:**
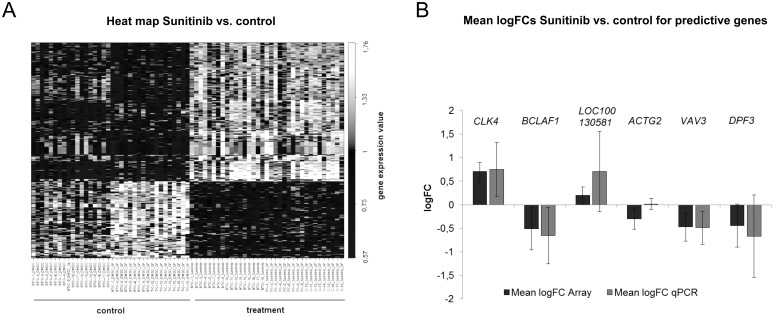
Heterogeneity of expression response to Sunitinib treatment. (A) Shown is a heat map of the 300 most differentially expressed genes when comparing Sunitinib treated with untreated samples. The samples are nicely separated into treated vs. untreated samples. However, the pronounced stripes in the heat map indicate that the vast majority of genes change expression only in subsets of cases. (B) The mean logFCs between control and Sunitinib treated samples for the predictive genes (CLK4, BCLAF1, LOC100130581, ACTG2, VAV3, DPF3) of 6 BTIC lines was calculated using (i) Microarray data and (ii) β-Actin normalized expression values assessed by qPCR for each individual gene.

All data analysis was restricted to the 500 genes with the highest expression variances across all samples. Notably, the expression differences between BTICs from different patients were stronger than the differences we observed in the identical cultures before and after treatment (Fig. S4). In order to zoom in on treatment effects, we compensated for inter-tumor variability computationally using the batch effect correction algorithm *Combat* [35]. Fig. 4A shows the 300 most regulated genes in response to Sunitinib treatment (FDR<0.001). Clearly, the samples are nicely separated into treated vs. untreated samples. However, the pronounced stripes in the heat map indicate that the majority of genes change expression only in subsets of cases. This result is well expected given the heterogeneous response of the pathways that ultimately shape these expression profiles.

### Gene expression profiles of treated but not of untreated BTIC lines predict proliferation

We proceeded to develop gene expression signatures to predict proliferation 96 hours after treatment. We derived these signatures from (a) expression levels prior to treatment and (b) expression levels 6 hours after treatment. Our signatures did not only aim to distinguish responders from non-responders (classification) but aimed to predict the XXT assay based proliferation rates quantitatively (regression). We used *least angle regression* [38] to simultaneously identify a set of signature genes, the optimal number of signature genes, and weights for the chosen genes. Proliferation 96 hours after treatment was predicted using the resulting weighted average of expression of the identified genes. Predictions were done in leave one out cross validation. Importantly, gene selection was done for every left out sample separately to avoid overly optimistic results due to overfitting. The x-axis shows cross-validated predictions of proliferation response after 96 hours based on gene expression levels monitored 6 hours after treatment, while the y-axis shows the actual proliferation measurements after 96 hours. The correlation between predicted and measured proliferation is significant (p<0.01, chi-square test) (Fig. 2D). When using the complete data set without leaving out samples the same algorithm identifies a 6-gene signature (CLK4, BCLAF1, LOC100130581, ACTG2, VAV3, DPF3) that can be used to predict proliferation of BTIC lines in independent samples.

Hence, early expression changes in response to treatment forecast long-term functional responses. In contrast, we were not able to predict proliferation from expression data that was recorded prior to treatment using the exact same statistical analysis strategy (p = 0.98; Fig. 2E.). In addition we did not observe any correlation between *in vitro* response and the strength of signaling modulation after treatment that was analyzed concurrently with gene expression profiles (Fig. S5). Consequently, semi-quantitative Western blot analysis of a selected panel of signaling transmitter molecules did not provide any information for the prediction of treatment effects.

To verify the gene expression levels of the 6 signature genes measured with microarray, we assessed the expression levels in 6 of the BTIC lines by qPCR analysis (Fig. 4B). The qPCR results confirmed that the directions of regulation were consistent with those observed by microarray analysis. We used β-Actin normalized expression values. Fig. 4B shows that the observed log fold changes were well reproduced for (5/6) signature genes using qPCR. The exception is ACTG2. Of note, ACTG2 has the smallest weight of all genes in the signature. Its weight is 3 orders of magnitude smaller then e.g. the weight of DPF3 (data not shown). In summary, prediction of proliferation response is possible. However, BTIC lines had to be confronted with the drug to make them release predictive information.

### Gene expression profiles of treated and untreated BTIC lines do not predict migration

Since inhibition of proliferation did not correlate with inhibition of cellular motility, we aimed to develop a different gene expression signature to predict motility. Using the same statistical strategy as described under proliferation prediction, we could not identify a gene signature that predicted migration after a 16 hours incubation period (Fig. 3D, E).

## Discussion

*In vitro* drug testing tools for predicting treatment effects in tumor patients were undertaken for more than 30 years. None of them has reached clinical application. Limitations lay in the long-term culture of differentiated tumor cell lines with prolonged treatment periods and conventional assays as readout. It is well known that tumor lines propagated under serum-containing conditions exert alterations in their molecular phenotype [43]. Additionally, the use of serum additives induces *ex vivo* modifications of functional behavior making valuable results unlikely. Finally, treatment response prediction before *in vivo* treatment was not assessed up to now using -omics technologies. To approach this, we first used serum-free short-term cultures of freshly resected high-grade gliomas; second, we performed short-term treatment with Sunitinib in a dose correlating to *in vivo* drug levels; and third, we monitored the response of thousands of molecular variables assessed by a microarray to define a genetic pattern of response.

We show that a gene signature deduced from microarray can predict inhibition of proliferation by Sunitinib in short-term serum-free cultures of brain tumor initiating cells (BTIC). In contrast the transcriptomes prior to treatment did not allow for the prediction of treatment response explaining the failure of previous attempts to establish predictive gene expression signatures in the field of glioma.

It is important to note that our signature is truly predictive. We monitored gene expression 6 hours after start of treatment. Considering an average cell cycle time of around 20 hours, we therefore forecast proliferation inhibition detected 96 hours after treatment from observations recorded only 6 hours after treatment. Interestingly, not the activation of individual pathways assessed by Western blots but the gene expression signature identified by microarrays allowed the prediction of treatment response. We therefore speculate that multiple alternative pathway activation constellations can lead to the same downstream result: the typical expression signature we observed if treatment impaired proliferation.

Tumor growth, progression and metastasis are partly mediated by activated receptor tyrosine kinases [44]. Complex signaling cross-talks between different growth-factor cascades regulate the self renewal and invasive capacity of BTICs [45]. Sunitinib inhibits selective VEGF/PDGF-stimulated activation of STAT3, AKT and ERK1/2 in BTIC cultures. Interestingly, we observed heterogeneous modulation of phosphorylation when Sunitinib was compared to control treatment with and without exogenous VEGF/PDGF stimulation. Tumor cells acquire genomic alterations that greatly reduce their dependency on exogenous growth stimulation, conserving their proliferation, survival and motility [46], [47]. In our assays, we did not notice any morphological changes that would indicate differentiation during culture in VEGF/PDGF restricted medium, and we proved that PDGF/VEGF preserves constant proliferation capacity of BTICs. Therefore we are confident that our experimental conditions allow the detection of the maximum effect of Sunitinib on essential signaling pathways and presumably also on transcription.

The intracellular signaling network is highly complex and an analysis of signaling pathway modulation can only provide a small insight. This may be one reason that particular transmitters cannot predict functional effects *in vitro*. As an example, it has been reported that short-term treatment with Sunitinib induces the expression of the dual lipid and phosphatase PTEN that negatively regulates PI3K/AKT [12], whereas at long-term exposure induces epigenetic silencing of the PTEN gene [48]. Therefore, regulation of pAKT after 6 hours of treatment may differ from pAKT after 96 hours and thus may not predict impaired cell growth after 96 hours. This observation corresponds well to other findings in the literature. Yang et al. [10] showed that phosphorylated ERK1/2 was not affected, but AKT and STAT3 phosphorylation was substantially reduced in medulloblastoma cell lines after short-term treatment with Sunitinib. Furthermore, in line with the results of Zhou et al. [49], differences of pERK were not statistically relevant to distinguish between Sunitinib sensitive and resistant U87MG glioma xenograft tumors.

Cellular viability testing using the XTT-assay revealed that BTICs differ according to their response to Sunitinib. This corresponds well to clinical data where only a small subgroup of patients with high-grade gliomas responded to treatment with Suntininib. Only in a small subset of Sunitinib treated BTIC cultures, we found a reduction of viable cells down to 56% compared to control treatment. As we did not observe cytotoxic short-term effects of 1 µM Sunitinib, the decrease of viable cells is likely to be a consequence of a reduced proliferation rate. Here, the correlation between predicted growth inhibition and measured growth inhibition was highly significant. Inhibition of cellular growth at clinically relevant concentrations observed in other studies using *in vitro* models are comparable to those detected in our study [11], [13], [14].

Concerning our migration assays, a steep onset of migration and a halted volumetric growth of spheroids during the first 24 hours has been published elsewhere [50]. Therefore, the contribution of proliferation in this experimental setup can probably be neglected. The effect of Sunitinib on migration was independent of the BTIC line specific motility and again very heterogeneous among the whole BTIC panel.

Based on our results, we suggest a novel design for predictive gene expression studies. We argue that it is important to ask the right cells the right questions. Here, untreated bulk tumor cells might not hold the necessary predictive information. Progenitor cells selected by short-term *in vitro* culture and treated over short periods of time, in contrast, show patterns of treatment response. We can only speculate why only expression signatures after treatment hold prognostic information. If the response is not stochastic, there must be a molecular difference between the two cell types, most likely genetic or epigenetic modifications, that alter the way incoming signals (treatment) are processed by the cells. This difference may only be reflected in expression profiles after challenging the cells with treatment, as they only then activate a defence response, which then leaves traces in gene expression profiles. *In vitro* cultures are a valid tool to confront cells with an agent before expression profiling. Our preclinical study shows that this strategy yields information that is not accessible from biopsy profiles. While this does not proof that our strategy will be useful in clinical trails, it is encouraging as a first development step.

## Supporting Information

Figure S1 **Comparison of proliferation regulation with XTT-assay against BrdU incorporation assay.** Proliferation assays was performed as described earlier. Cells were treated with 0.2 µM, 1 µM Sunitinib or 0.00025% DMSO for 120 hours. For both assays the mean absorbance of Sunitinib treated cells relative to control cells were calculated and depicted as bar graphs. Almost identical results were obtained with XTT-Assay and BrdU incorporation assay for the two representative cell lines BTIC-1 and BTIC-2.
(TIF)

Figure S2 **Comparison of BTIC proliferation with different growth factor supplementation.** Proliferation assays were performed as described in the material and methods section. Media containing 25 ng/ml of each bFGF and EGF or 25 ng/ml of each VEGF and PDGF-AB was added instead of treatment. The XTT proliferation assay was evaluated after 48, 96, and 144 hours. No significant difference of proliferation could be shown at 48 and 144 hours cultivation under defined conditions (p = 0.056 and 0.1, respectively).
(TIF)

Figure S3 **Verification of the spheroid expansion assay with the** ***in vitro*** **scratch assay.** Scratch migration assays were performed as described above with 1 µM Sunitinib or 0.00025% DMSO for 24 hours. (A) Sunitinib induced a significant decrease of migration in comparison to controls. (B) No significant difference of inhibition of migration could be shown in spheroid vs. scratch assays, verifying spheroid assay results (p = 0.116).
(TIF)

Figure S4 **Correction for BTIC line specific expression variances enables the detection of treatment specific expression variances.** (A) The 500 most variable genes were hierarchically clustered according to Euclidean distances showing that all treatment samples cluster within the corresponding BTIC line. (B) After compensation for inter-tumoral variability using the batch effect correction algorithm *Combat* samples clustered mainly within treatment specific groups.
(TIF)

Figure S5 **Mitogenic signaling modulation does not correlate to proliferation or migration inhibition after treatment.** For each BTIC line XTT absorbance relative to control (A, B, C) or the Spheroid size relative to control (D, E, F) is plotted against the semi-quantitative consensus strength of phosphorylation specific Western blot signals for one of the 3 (AKT, ERK, STAT3) signaling molecules. None of the phosphorylation levels of the signal transducers correlated to inhibition of proliferation or migration.
(TIF)

Table S1 **Clinical and biological information of parental tumor specimen from the analyzed BTIC panel vs. BTIC lines.** Histology and WHO tumor grade were evaluated in the original tumors by an independent neuropathologist (MR). MGMT = Methyl-Guanine-Methyl-Transferase; meth. = methylated MGMT-Promotor (>8%); unmeth. = unmethylated MGMT-Promotor; IDH = isocitrate dehydrogenase; wt = wild type; n.d. = not determined; f = female; m = male; R = Radiotherapy 60 Gy; RC = Radiotherapy 60 Gy plus Chemotherapy with Temozolomide 75 mg/m^2^ daily during radiotherapy, then adjuvant Temozolomide 150–200 mg/m^2^ d1-5/28 days (Stupp protocol).
(DOCX)

Table S2 **RT-PCR primer sequences.** Primers for the 6-gene signature set (CLK4, BCLAF1, LOC100130581, ACTG2, VAV3, DPF3) that can be used to predict proliferation of BTIC lines in independent samples are given.
(DOCX)
