# Supplementary figures and images for: Response-Predictive Gene Expression Profiling of Glioma Progenitor Cells In Vitro
Source: PLoS One. 2014 Sep 30;9(9):e108632. doi: 10.1371/journal.pone.0108632 (PMC4182559; doi:10.1371/journal.pone.0108632)

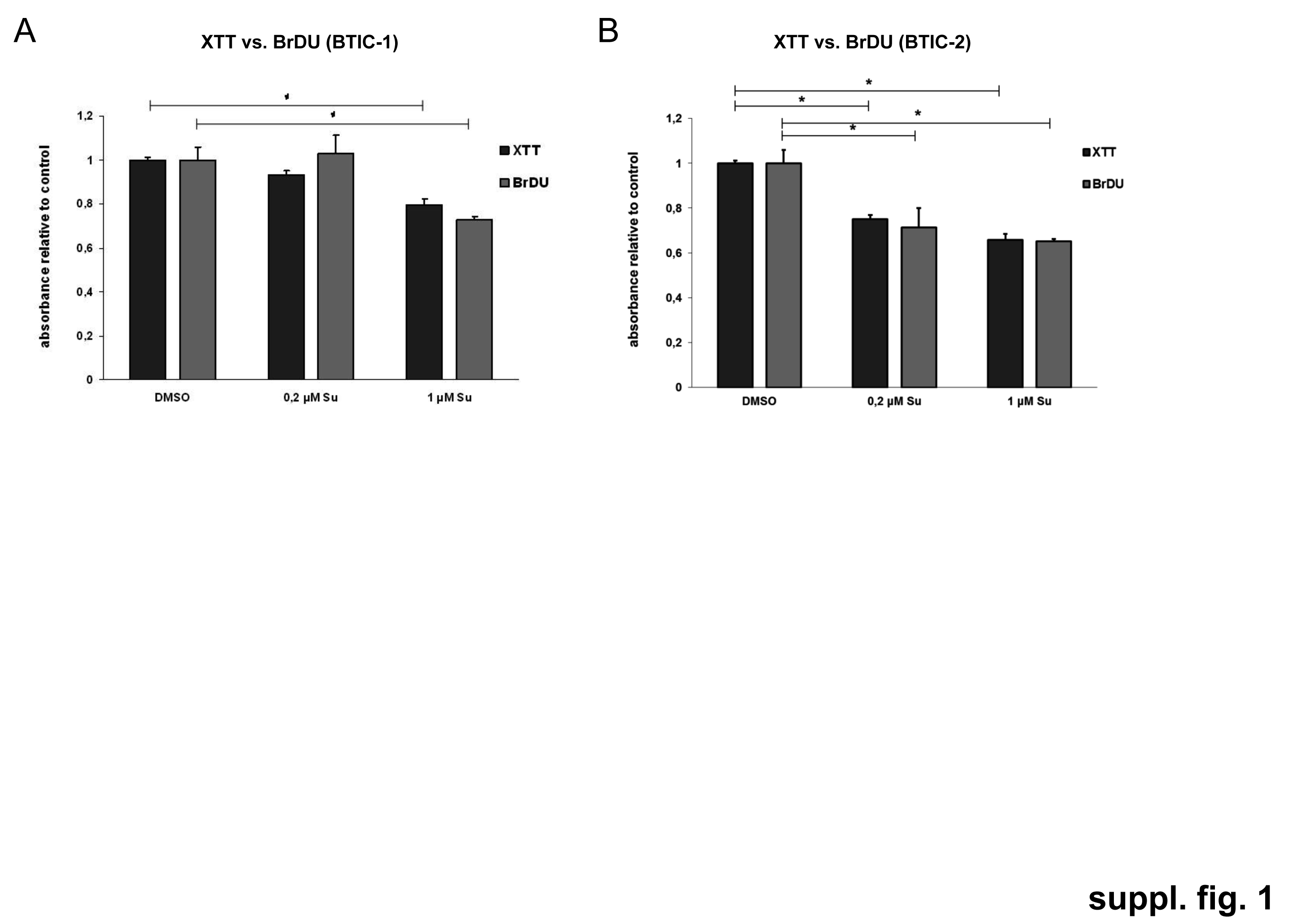

Supplement: Figure S1 — Comparison of proliferation regulation with XTT-assay against BrdU incorporation assay. Proliferation assays was performed as described earlier. Cells were treated with 0.2 µM, 1 µM Sunitinib or 0.00025% DMSO for 120 hours. For both assays the mean absorbance of Sunitinib treated cells relative to control cells were calculated and depicted as bar graphs. Almost identical results were obtained with XTT-Assay and BrdU incorporation assay for the two representative cell lines BTIC-1 and BTIC-2. (TIF) [file pone.0108632.s001.tif]

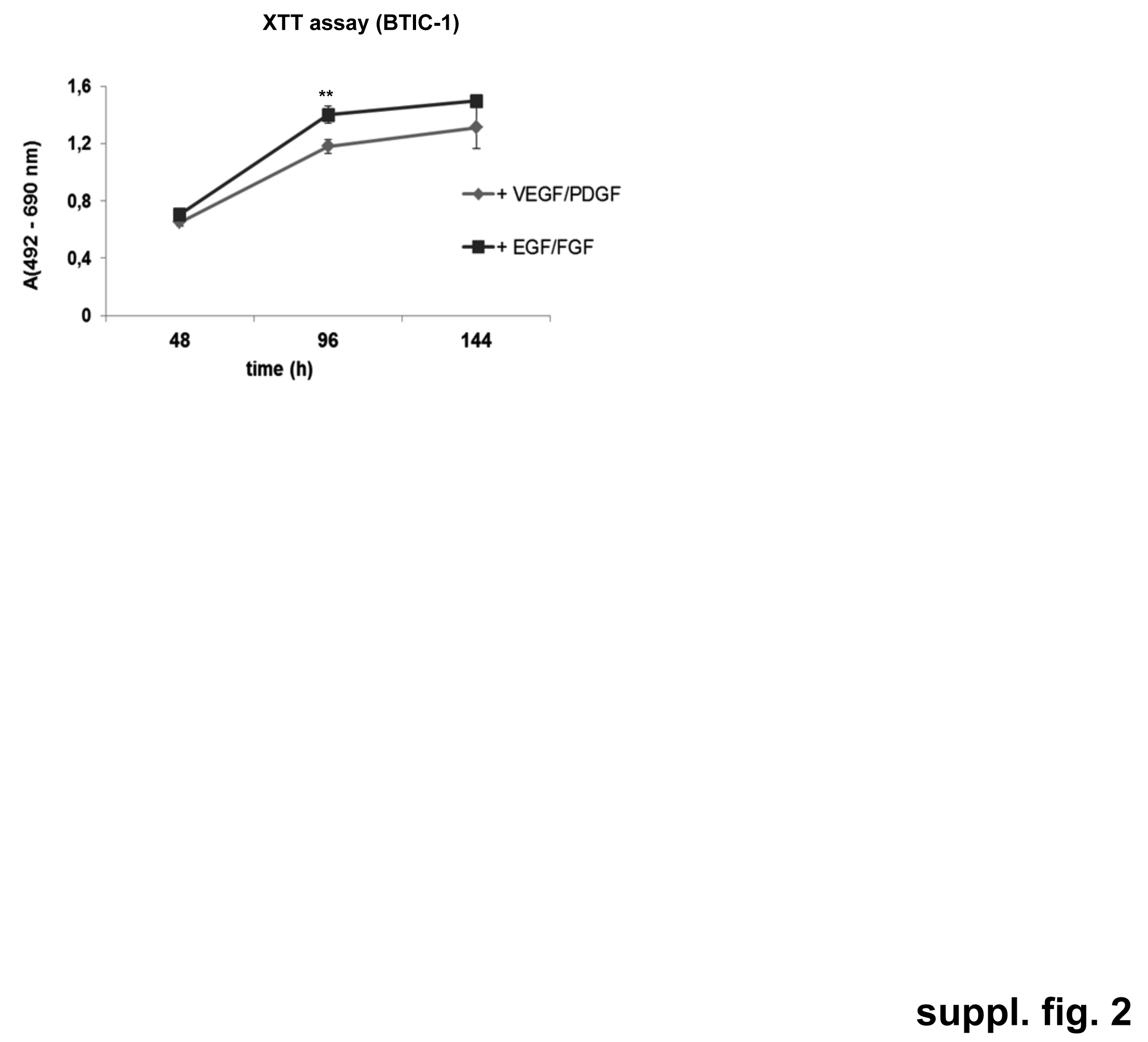

Supplement: Figure S2 — Comparison of BTIC proliferation with different growth factor supplementation. Proliferation assays were performed as described in the material and methods section. Media containing 25 ng/ml of each bFGF and EGF or 25 ng/ml of each VEGF and PDGF-AB was added instead of treatment. The XTT proliferation assay was evaluated after 48, 96, and 144 hours. No significant difference of proliferation could be shown at 48 and 144 hours cultivation under defined conditions (p = 0.056 and 0.1, respectively). (TIF) [file pone.0108632.s002.tif]

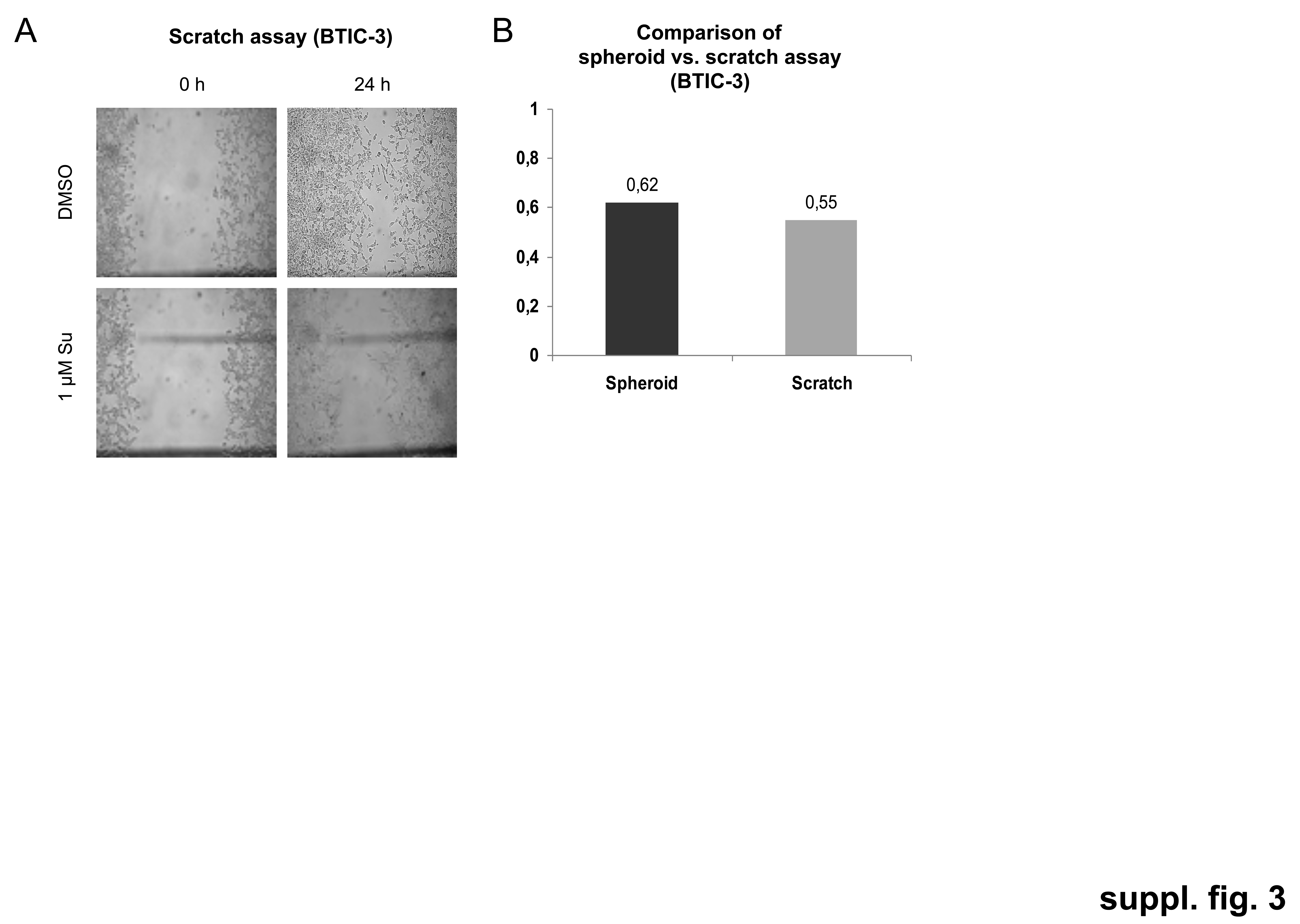

Supplement: Figure S3 — Verification of the spheroid expansion assay with the in vitro scratch assay. Scratch migration assays were performed as described above with 1 µM Sunitinib or 0.00025% DMSO for 24 hours. (A) Sunitinib induced a significant decrease of migration in comparison to controls. (B) No significant difference of inhibition of migration could be shown in spheroid vs. scratch assays, verifying spheroid assay results (p = 0.116). (TIF) [file pone.0108632.s003.tif]

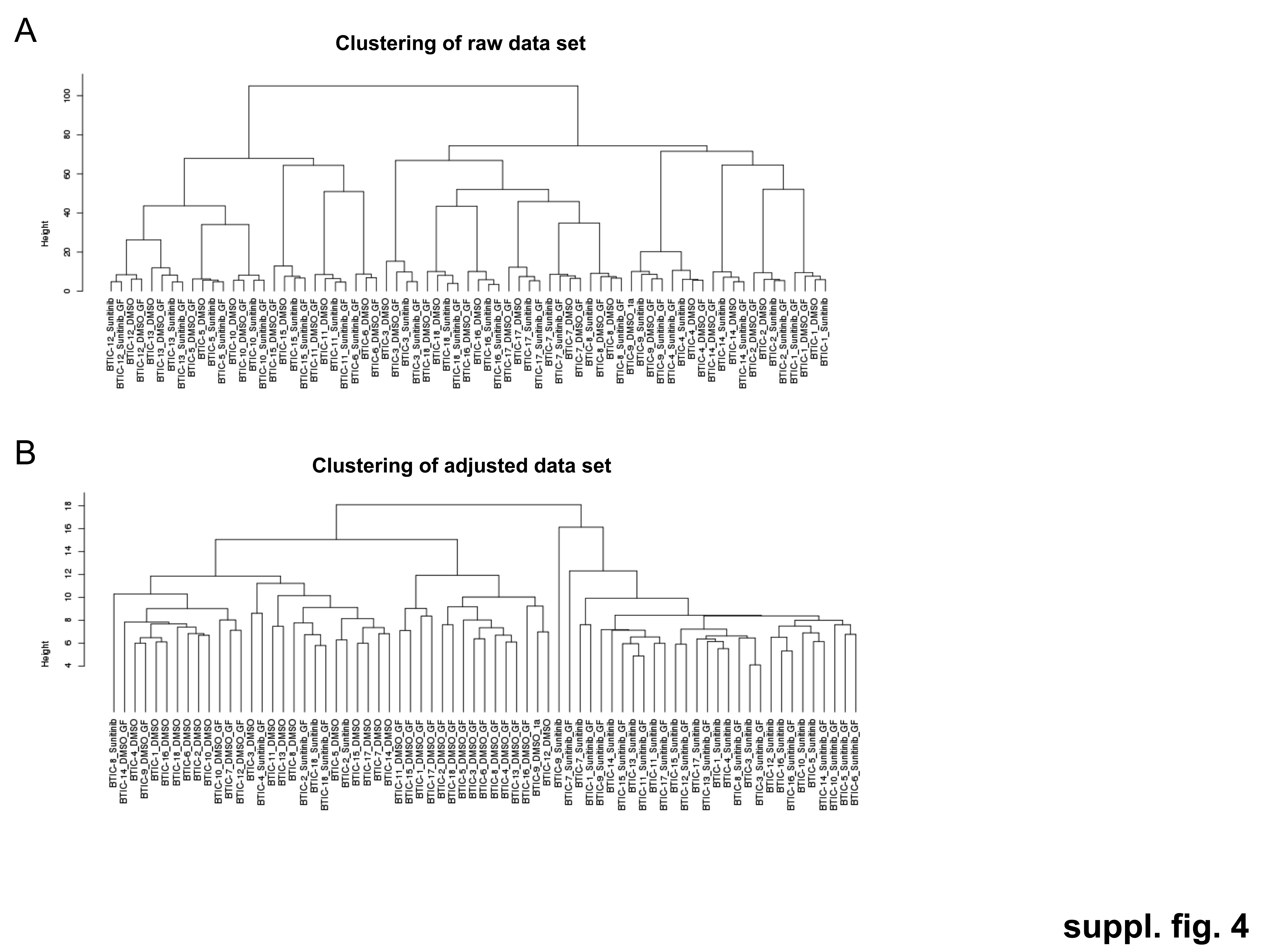

Supplement: Figure S4 — Correction for BTIC line specific expression variances enables the detection of treatment specific expression variances. (A) The 500 most variable genes were hierarchically clustered according to Euclidean distances showing that all treatment samples cluster within the corresponding BTIC line. (B) After compensation for inter-tumoral variability using the batch effect correction algorithm Combat samples clustered mainly within treatment specific groups. (TIF) [file pone.0108632.s004.tif]

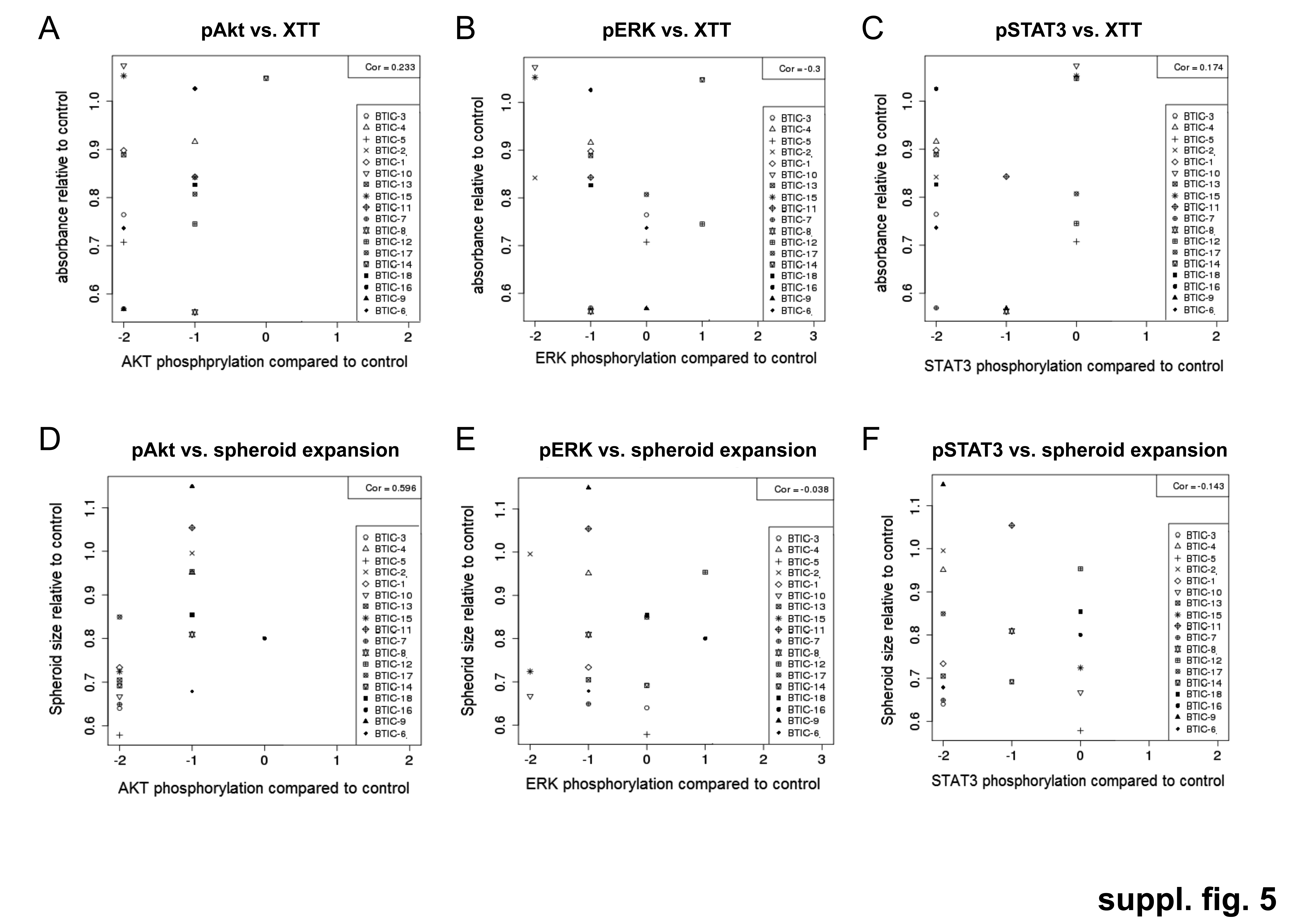

Supplement: Figure S5 — Mitogenic signaling modulation does not correlate to proliferation or migration inhibition after treatment. For each BTIC line XTT absorbance relative to control (A, B, C) or the Spheroid size relative to control (D, E, F) is plotted against the semi-quantitative consensus strength of phosphorylation specific Western blot signals for one of the 3 (AKT, ERK, STAT3) signaling molecules. None of the phosphorylation levels of the signal transducers correlated to inhibition of proliferation or migration. (TIF) [file pone.0108632.s005.tif]
