# Supplementary material for: Response-Predictive Gene Expression Profiling of Glioma Progenitor Cells In Vitro
Source: PLoS One. 2014 Sep 30;9(9):e108632. doi: 10.1371/journal.pone.0108632 (PMC4182559; doi:10.1371/journal.pone.0108632)
Supplement: Table S1 — Clinical and biological information of parental tumor specimen from the analyzed BTIC panel vs. BTIC lines. Histology and WHO tumor grade were evaluated in the original tumors by an independent neuropathologist (MR). MGMT = Methyl-Guanine-Methyl-Transferase; meth. = methylated MGMT-Promotor (>8%); unmeth. = unmethylated MGMT-Promotor; IDH = isocitrate dehydrogenase; wt = wild type; n.d. = not determined; f = female; m = male; R = Radiotherapy 60 Gy; RC = Radiotherapy 60 Gy plus Chemotherapy with Temozolomide 75 mg/m2 daily during radiotherapy, then adjuvant Temozolomide 150–200 mg/m2 d1-5/28 days (Stupp protocol). (DOCX) [file pone.0108632.s006.docx]

| **BTIC** | **Original tumor** | | | | **Patient characteristics** | | | | **Primary cell culture** | | | |
| --- | --- | --- | --- | --- | --- | --- | --- | --- | --- | --- | --- | --- |
|  | **Histology** | **WHO-Grade (I-IV)** | **MGMT-methyl.** | **IDH1 (wt/mut.)** | **Age at diagnosis** | **Gender** | **Prior Therapy** | **Survival (/weeks)** | ***in vitro* growth** | **MGMT-methyl.** | **CD133 pos. (/%)** | **Mol. classification** |
| **1** | GBM (prim.) | IV | n.d. | wt | 65 | m | R/C | 2 | adherent | meth. | 22 | proneural |
| **2** | GBM (prim.) | IV | meth. | wt | 67 | m | R/C | 35 | adherent | meth. | 7 | proneural |
| **3** | GBM (sec.) | IV | unmeth. | wt | 88 | m | R/C | 39 | adherent | unmeth. | 4 | proneural |
| **4** | GBM (prim.) | IV | n.d. | wt | 78 | f | R/C | n.a. | adherent | meth. | 26 | proneural |
| **5** | GBM (prim.) | IV | n.d. | n.d. | 56 | m | R/C | 32 | adherent | meth. | n.d. | mesenchymal |
| **6** | GBM (prim.) | IV | n.d. | n.d. | 44 | m | R/C | 75 | adherent | meth. | 0 | mesenchymal |
| **7** | GBM (prim.) | IV | unmeth. | wt | 52 | f | R/C | 65 | Spheres | unmeth. | 1 | proneural |
| **8** | GBM (prim.) | IV | unmeth. | wt | 52 | f | R/C | 16 | Spheres | unmeth. | 3 | proneural |
| **9** | AA (prim.) | III | unmeth. | p.R132H | 30 | m | R | 163 | adherent | meth. | 10 | n.d. |
| **10** | GBM (prim.) | IV | meth. | n.d. | 46 | m | R/C | 75 | adherent | meth. | 1 | mesenchymal |
| **11** | GS (prim.) | IV | meth. | wt | 55 | m | R/C | 70 | adherent | meth. | 6 | mesenchymal |
| **12** | GBM (prim.) | IV | meth. | wt | 69 | m | R/C | 66 | adherent | meth. | 29 | mesenchymal |
| **13** | GBM (sec.) | IV | unmeth. | p.R132H | 42 | m | R/C | 34 | adherent | meth. | 62 | mesenchymal |
| **14** | GBM (prim.) | IV | meth. | wt | 78 | f | R/C | 58 | adherent | meth. | 0 | mesenchymal |
| **15** | GBM (prim.) | IV | unmeth. | wt | 50 | f | R/C | 31 | Spheres | meth. | 3 | mesenchymal |
| **16** | GBM (prim.) | IV | unmeth. | wt | 72 | m | R/C | 55 | adherent | unmeth. | 6 | proneural |
| **17** | GBM (sec.) | IV | meth. | wt | 49 | f | R/C | 35 | Spheres | meth. | 35 | proneural |
| **18** | GBM (prim.) | IV | unmeth. | wt | 49 | m | R/C | 82 | adherent | unmeth. | 0 | proneural |
