# Supplementary material for: Response-Predictive Gene Expression Profiling of Glioma Progenitor Cells In Vitro
Source: PLoS One. 2014 Sep 30;9(9):e108632. doi: 10.1371/journal.pone.0108632 (PMC4182559; doi:10.1371/journal.pone.0108632)
Supplement: Table S2 — RT-PCR primer sequences. Primers for the 6-gene signature set (CLK4, BCLAF1, LOC100130581, ACTG2, VAV3, DPF3) that can be used to predict proliferation of BTIC lines in independent samples are given. (DOCX) [file pone.0108632.s007.docx]

| **CLK4** | Forward: TGGACACTTTGGGTGAAGGA  Reverse: CTGGACACATCGGAAGACACT |
| --- | --- |
| **BCLAF1** | Forward: CGCGTCGAAGGTAGCTCTAT  Reverse: TTGGAGCGACCCATTTCTTTT |
| **ACTG2** | Forward: AGCCTTCCTTTATTGGCATGGA  Reverse: GTGGAGAGAGAGGCCAGGAT |
| **VAV3** | Forward: GAGAAGTAAATGGCAGGGTGG  Reverse: GCTAAGGAGGGAGGATGTTGA |
| **DPF3** | Forward: AGCGTGCGTCTTCCCTT  Reverse: GCTCCACTTCAGGTTTTATCTCC |
| **LOC100130581** | Forward: AGCCAGAGGAGGAATGGAAC  Reverse: ATACCGAAGCACCTGAGCAAC |
| **Aktin** | Forward: TGGCACCACACCTTCTACAAT  Reverse: AGAGGCGTACAGGGATAGCA |
